# Chemical composition and bioinsecticidal activity of bioinputs produced by *Saccharopolyspora spinosa*

**DOI:** 10.1007/s00253-025-13655-3

**Published:** 2025-12-11

**Authors:** Luís Felipe Rodrigues Costa, Amanda Evelyn Miranda, Márliton Pereira dos Santos, Jorge Luiz Soares dos Anjos, Fernanda Menezes Maia, Tiara da Costa Silva, Mario Machado Martins, Joseilton Faria Silva, Isac Pereira Soares Martins, Clarice Diniz Alvarenga, Marlon Cristian Toledo Pereira, Nelson de Abreu Delvaux Júnior, Luciano Pereira Rodrigues

**Affiliations:** 1 Department of Agricultural Sciences, State University of Montes Claros, Rua Reinaldo Viana, 2630, Janaúba, MG 39448-581 Brazil; 2 Institute of Engineering, Science and Technology, Federal University of the Jequitinhonha and Mucuri Valleys, Av. Um, 4.050, Janaúba, MG 39447-790 Brazil; 3 Institute of Chemistry, Federal University of Uberlândia, Av. João Naves de Ávila, 2121, Uberlândia, MG 38400-902 Brazil; 4 Institute of Biotechnology, Federal University of Uberlândia, Rua Acre, 1004, Bloco 2E, Uberlândia, MG 38405-319 Brazil; 5 Institute of Exact, Natural Sciences and Education, Federal University of Triângulo Mineiro, Av. Randolfo Borges Júnior, 1400, Uberaba, MG 38025-180 Brazil

**Keywords:** Secondary metabolites, Spinosyns, *Spodoptera frugiperda*, *Dalbulus maidis*, *Ceratitis capitata*

## Abstract

**Abstract:**

The growing demand for sustainable agricultural pest control solutions has increased the use of microbial bioinput. This study characterized an industrial bioinput produced by *Saccharopolyspora spinosa*, focusing on identifying metabolites and quantifying spinosyns A and D by HPLC-MS. The concentrations of these compounds were 29.4 ± 3.6 mg L^−1^ and 13.3 ± 1.5 mg L^−1^, respectively, for a total of 42.7 mg L^−1^. The limit of quantification (LOQ) and limit of detection (LOD) values obtained were 13.7 ng L^−1^ and 4.16 ng L^−1^ for spinosyn A and 7.63 ng L^−1^ and 2.31 ng L^−1^ for spinosyn D, respectively. The bioinput contained essential amino acids (leucine, phenylalanine, and tryptophan), plant cell death inducers (sphinganine), diketopiperazines with insecticidal potential (Cyclo(Met-Val)), larvicides (*N*-stearoyl tryptophan), antimicrobials (Penicitrinol D), and phospholipids associated with cell defense and stress responses (phosphatidylethanolamine, phosphatidylserine, ceramides, and mycolactones). *In vitro* trials demonstrated mortalities of 82.5% for *Spodoptera frugiperda*, 100.0% for *Dalbulus maidis*, and 64.1% for *Ceratitis capitata*. These results are statistically equivalent to those obtained using commercial products, which cost up to five times more. This bioinput’s chemical diversity suggests multifunctional action, probable synergism, and a lower risk of resistance, which reinforces its potential in biological agricultural management.

**Key points:**

• *Identification of a diverse set of bioactive compounds, including spinosyns A and D.*

• *Bioinput achieves statistical efficacy equal to commercial products in in vitro tests.*

• *A sustainable, cost-effective alternative for large-scale production and application.*

**Supplementary information:**

The online version contains supplementary material available at 10.1007/s00253-025-13655-3.

## Introduction

Biopesticides have emerged as attractive and environmentally friendly bioinput, enhanced for the management of diseases and pest infestations. The demand for organically grown food has increased worldwide and, consequently, so has the demand for bioinput due to health, safety, and environmental concerns (Kumar et al. 2023). Biological control of soil-borne plant pathogens is a potential alternative to the use of chemical pesticides, which have been proven to be harmful to the environment and public health (Chet and Inbar 1994).

So-called biopesticides include viruses, bacteria, fungi, predators, parasitoids, and pheromones that exhibit a variety of modes of action. They are less toxic, rapidly degradable, and more targeted to specific pests. The effectiveness of any bioinput with the ability to act as a biopesticide is determined by a variety of factors, such as the source of origin, formulation, application system and dosage, toxicity to the target pest, and impact on non-target organisms, including animals and humans (Hezakiel et al. 2024).

Spinosyns are active substances produced through chemical synthesis or fermentation processes using mainly the Actinobacteria *Saccharopolyspora spinosa* and constitute a class of broad-spectrum insecticides against many pests harmful to a wide variety of agricultural crops (Bacci et al. 2016). In small doses, spinosyns are effective in controlling pests in the field and post-harvest. High-performance bioinput obtained by cell multiplication of *S. spinosa* presents the analogues spinosyn A and D as secondary metabolites, which, in addition to being considered low environmental impact, are less aggressive against non-target species. Furthermore, their phytotoxicity in mammals is low and they degrade rapidly in various environmental matrices (Cleveland et al. 2002). The *S. spinosa* is the exclusive natural producer of the insecticidal compounds, spinosyns A and D, as this actinomycete is the only species identified that possesses the complex polyketide synthase (PKS) gene cluster responsible for the synthesis of the unique tetracyclic macrolide structure of spinosyns (Mertz and Yao 1990; Thompson et al. 2000). Additionally, the use of *S. spinosa* is central to modern agricultural biotechnology, being the focus of intense studies on metabolic engineering and bioprocess optimization to increase Spinosad yield (Jin et al. 2009; Yang et al. 2014).

Spinosyns act as activators of allosteric acetylcholine receptors and, thanks to their selective mode of action, resistance phenomena are uncommon, although some cases have recently been reported for some insect species, including the caterpillar *Spodoptera exigua* (Hübner) (Zuo et al. 2020; 2022) and the mosquito *Culex quinquefasciatus Say* (Hafez and Abbas 2021).

Spinosyns J and L, obtained preferentially by synthetic routes, are marketed under the name Spinetoram and are commonly used against *Anastrepha fraterculus* (Wiedemann), known as the South American fruit fly (Schütze et al. 2018). Spinetoram is also effective in controlling six beetles commonly found in stored grains, including *Sitophilus oryzae* (L.), *Rhyzopertha dominica* (Fabricius), *Prostephanus truncatus* (Horn), *Tribolium confusum DuVal*, *Sitophilus granarius* (L.), and *Oryzaephilus surinamensis* (L.) (Vassilakos and Athanassiou et al. 2012). Spinetoram has also been tested and is currently widely used to control *Anthonomus grandis* Boheman, commonly known as the cotton boll weevil (Rolim et al. 2019). Spinosyns A and D, marketed under the name Spinosad, were combined with spinosyns J and L to control beetles such as *S. oryzae* and *Tribolium castaneum* (Herbst) in their adult stage in stored grains (Azab 2015).

Controlling *S. frugiperda* is particularly challenging due to the high selection pressure of insecticides, requiring up to six applications per crop cycle in some regions. Studies show a decline in the effectiveness of chemical products as the caterpillar develops, with average mortality declining from 98.5% in the second instar to 80.1% in the fifth instar. This reduction in control efficacy of more advanced instars, combined with concerns about selectivity and the development of resistance, reinforces the need for biological alternatives, such as spinosyn-based bioinputs, that maintain high efficacy and selectivity (Gonçalves et al. 1997). Spinosad, at a dose of 24 g a.i. ha^−1^, proved to be a fast-acting and highly effective insecticide in the initial control of *S. frugiperda*, achieving a satisfactory control level of 84% just 3 days after application. This performance was superior to other chemical groups, such as the benzoylureas, which only reached efficacy after the seventh day, and comparable to high-performance carbamates, with 90% efficacy in the same period (Tomquelski and Martins 2007).

The corn leafhopper, *Dalbulus maidis*, represents a growing challenge in maize management due to its capacity to vector pathogens, such as *Spiroplasma kunkelii*, which causes corn stunt disease (pale stunt). Although seed treatment is effective in the early stages of the crop, research demonstrates that insecticides applied via foliar spray exhibit low efficacy in controlling *D. maidis* adults, despite being able to reduce the insect’s feeding and the pathogen’s transmission rate. This limitation in the field control of adults reinforces the need to search for bioinputs with innovative or alternative mechanisms of action for the integrated management of this key pest (Yamamoto et al. 2020).

In the management of the *Ceratitis capitata*, toxic baits represent a fundamental strategy, particularly during the pre-harvest period. Spinosad and Spinetoram have proven to be promising ingredients, as studies indicate a mortality rate exceeding 80% in adults, making them effective alternatives to organophosphate insecticides. Toxic baits formulated with Spinetoram at 80% of the manufacturer’s recommended concentration achieved an LT_50_ after 6 h (Baronio et al. 2019).

Despite their effectiveness in pest control, some biotic and abiotic factors can interfere with the mortality of insects treated with spinosyns, including the target species, temperature, relative humidity, and insecticide dosage (Vassilakos 2023).

Genetic engineering techniques are being developed to modify *S. spinosa* and increase the production of spinosyns A and D. Studies duplicating the genes involved in the initial stages of deoxyglucose biosynthesis have proven effective in significantly increasing spinosyn yield (Madduri et al. 2001a, 2001b).

Another study involving recombinant genomic shuffling resulted in an increase of more than 200% in spinosyns A and D, reaching a level of 547 mg L^−1^ using mini-reactors with a volumetric capacity of 5 L (Jin et al. 2009). A rational metabolic engineering study of *S. spinosa* to improve the production of spinosyns A and D was efficient and reached values of 372 and 217 mg L^−1^, respectively (Jha et al. 2014).

The biotechnological process for obtaining bioinput requires, in addition to the genetic optimization of *S. spinosa* strains, adequate conditions of pH, temperature, substrate, and high-quality control to avoid cross-contamination through water, air, and the surface of the bioreactors (Faria et al. 2023; Rocha et al. 2024). Of these, the medium in which the microorganisms develop, as well as the substrate, is the parameter that should preferably be optimized to maximize the production of spinosyns A and D. A new macrolide producer was cultivated in a variety of media to optimize the concentration of spinosyns in Spinosad (Madduri et al. 2001a, b). Statistical and regressive modeling methods were used to investigate the effects of the carbon source to optimize the average production of spinosyns. In this study, when the conventional glucose substrate was replaced by a mixture of mannitol, cottonseed meal, and corn maceration liquor as a carbon source, a 77.13% increase in the content of spinosyns A and D was obtained (Yang et al. 2016). The insecticidal activity of crude butenyl-spinosyn against *Thrips palmi Karny*, aphids, and *Plutella xylostella* (L.) insects was investigated, and the LC_50_ was determined to be 1.92 mg L^−1^, 1.23 mg L^−1^, and 0.27 mg L^−1^, respectively (Xu et al. 2024).

The identification and quantification of secondary metabolites produced by *Saccharopolyspora spinosa*, especially spinosyns, are fundamental to ensure control efficacy, reduce costs, minimize resistance selection, and enable its expansion in integrated pest management. In this context, the bioinput inputs produced maximize product safety and quality, in addition to enabling their standardization, meeting legal requirements, and contributing to the development of more efficient and sustainable agricultural solutions (Chen et al. 2015; Huang et al. 2009).

This study has three main objectives: (i) to qualitatively evaluate, by high performance liquid chromatography coupled to mass spectrometry (HPLC-MS), the chemical composition of the bioinput produced on a large scale via fermentation with *S. spinosa*; (ii) to quantify, also by HPLC-MS, the levels of spinosyns A and D in bioinput produced by *S. spinosa* on a macroscale; and (iii) to analyze *in vitro* the effects of this bioinput on three insect pests of economic importance in Brazilian agriculture: *Spodoptera frugiperda* (J. E. Smith), *Dalbulus maidis* (DeLong & Wolcott), and *Ceratitis capitata* (Wiedemann).

## Material and methods

### Reagents and solutions

Fermentation process: peptone and yeast extract (Kasvi), amino acids, monosaccharides, disaccharides, polysaccharides, flours, and minerals (ACS científica). Chemical assays: acetonitrile (Carlo Herba) and formic acid (Dinâmica)-analytical grade. Bioassays: No reagents were used. For the experimental part and preparation of the solutions, Type 1 ultrapure water obtained from a Barnstead system was used. All analyses were performed in triplicate.

### Large-scale bioinput production by fermentation through cell multiplication of *Saccharopolyspora spinosa*

The macroscale production of the bioinput in this study utilized the actinomycete *Saccharopolyspora spinosa*. The choice of this strain is crucial because the evaluation focuses on the bioinput, which is a product derived directly from the macroscale fermentation process that can be carried out at any production site (on farm), and is not restricted only to a purified chemical product. The *S. spinosa* determines the final chemical profile of the bioinput, including the active proportion of spinosyn A and D and other secondary metabolites, which collectively define its total insecticidal activity (Thompson et al. 2000).

In the previously cleaned and sanitized bioreactor, 800 L of double-filtered water, activated carbon, and a bec clean filter were added. Subsequently, the pH and temperature of the water were adjusted to 7.0 and 22.0 °C, respectively, to ensure greater effectiveness of the ozonation system for water decontamination in the subsequent stage. Subsequently, the specific culture medium for the bacterium *S. spinosa*, supplied by the BioWorld group, was added at a concentration of 50 g L^−1^. Next, the pH and temperature of the mixture were adjusted to 7.5 and 29 °C, respectively, and it was subjected to the ozonation process for 10 min. After ozonation, we waited 35 min to perform the inoculation with *S. spinosa*, applied at a concentration of 0.5% (v v^−1^). During 120 h of agitation in the bioreactor, the bioinput produced is monitored for pH, temperature, and brix parameters. At the end, the bioinput was bottled, and the appropriate samples were collected and refrigerated between 7 and 10 °C for analysis.

Similar procedures were developed for bioinputs produced using other entomopathogenic microorganisms, bacteria: *Bacillus thuringiensis* (culture medium at a concentration of 30 g L^−1^, temperature of 32 °C, and multiplication time of 72 h) and *Chromobacterium subtsugae* (culture medium at a concentration of 20 g L^−1^, temperature of 26 °C, and multiplication time of 48 h), fungi: *Cordyceps fumosorosea* and *Beauveria bassiana* (culture medium at a concentration of 30 g L^−1^, pH of 4–5, temperature of 23 °C, and multiplication time of 72 h). All bacterial strains used in this experiment are coded by the American Type Culture Collection (ATCC) as follows: *S. spinosa* (ATCC-49460), *Bacillus thuringiensis* (ATCC-10792), and *Chromobacterium subtsugae* (ATCC-31532TM). Fungal strains are coded by the André Tosselo Tropical Culture Collection (ATTCC) as *Cordyceps fumosorosea* (ATTCC-4530) and *Beauveria bassiana* (ATTCC-4641).

### High performance liquid chromatography coupled to mass spectrometry (HPLC-MS)

Samples of bioinput produced on macroscale by *S. spinosa* were analyzed by HPLC-MS to identify the chemical composition and quantify spinosyns A and D at the Nanobiotechnology Laboratory at the Federal University of Uberlândia, Uberlândia-MG.

#### Determination of chemical composition

The chemical constituents were extracted using 500 µL of the bioinput samples and 1000 µL of spectroscopic-grade acetonitrile. The mixture was vortexed (Kasvi, K40-1028) for 5 min at 1300 rpm. It was then centrifuged (Eppendorf, 5430R) for 15 min at 10,000 rpm. The supernatant was collected and dried in a speed vac (GeneVac, DUC-22060-C00). Samples of the dried material were resuspended in 1000 µL of acetonitrile and 200 µL of water and filtered through 0.22-µm nylon filters.

The samples were injected into a liquid chromatograph (Agilent Infinity 1260) coupled to a high-resolution Q-TOF mass spectrometer (Agilent 6520 B), with an electrospray ionization (ESI) source in positive and negative modes. The mobile phase consisted of water acidified with 0.1% formic acid (A) and spectroscopic-grade acetonitrile (B). The elution gradient was 2% B (0 min), 98% B (0–10 min), and 98% B (10–17 min). The injection volume was 3 µL, and the sample was kept at 20 °C.

Chromatographic separation was performed at a rate of 400 µL min^−1^ using an Agilent Infinity Lab Poroshell HPH-C18 column (2.1 mm × 50 mm, 2.7 µm) with the oven at 30 °C. The ionization parameters used were nebulizer pressure of 58 psi, drying gas flow rate of 8 L min^−1^ at 220 °C, and capillary voltage of 4.5 kVa.

In the Biochemistry and Analytical Chemistry Laboratory at the State University of Montes Claros, Janaúba-MG, mass spectrometry (MS) analyses were performed, and the data were processed using MassHunter® software, which proposed molecular formulas for the compounds based on high-resolution *m/z* values. The Qualitative Analysis MassHunter and Molecular Structure Correlator programs were used to view the retention time of the compounds and their respective molecular masses using the data files from the results acquired by HPLC-MS. These experimental data were cross-referenced with databases and information available in the scientific literature, such as PubChem, ChemSpider, ChEMBL, SciFinder, and CAS (Chemical Abstracts Service), allowing for a proposal to identify the chemical composition in the bioinput samples produced by *S. spinosa* after storage for 15, 90, and 180 days.

#### Quantification of spinosyns A and D

For quantification of spinosyns A and D, the same sample preparation described in item 2.3.1 was performed. However, for each sample, three tubes of dry extracted material were used, resuspended in 100 µL of water and 50 µL of acetonitrile, and filtered through 0.22-µm nylon filters. These samples were analyzed by high-resolution HPLC-MS under the same chromatographic conditions presented in item 2.3.1. After injecting the samples, standard solutions of spinosyn A and spinosyn D (Spinosad, Sigma-Aldrich) at different concentrations (250,000; 125,000; 62,500; 31,250; 15,625; 7812.5; 3906. 25; 1953.12; 976.56; 488.28; 244.14 ng mL^−1^) were also injected to construct the calibration curve. The spectra obtained were processed using Agilent MassHunter Quantitative Analysis software, which allowed the calibration curves for each compound to be plotted, obtaining the *R*^2^ values and the equation of the line for the quantification of spinosyns A and D in the samples. The samples were analyzed using the parameters defined by the analytical curve, such as the retention time of the compound and the *m/z*. Thus, it was possible to calculate the concentration of spinosyns A and D based on the area of the peaks corresponding to each standard present in the samples. For the calculations, a purity of 90% was considered for spinosyn A and 50% for spinosyn D. The limit of detection (LOD) was calculated using the formula adopted by the National Institute of Metrology, Quality and Technology (INMETRO/DOQ-CGCRE-008) by dividing the limit of quantification (LOQ) obtained as the lowest point on the calibration curve by a factor of 3.3.

### Insect bioassays

Bioassays to evaluate the effects of bioinsecticides on the insects *S. frugiperda* and *D. maidis* were conducted at the Plant Pathology Laboratory of the Terras Gerais Experimental Research Station, Lavras-MG, and for *C. capitata* at the Biological Control Laboratory at the State University of Montes Claros, Janaúba-MG. Specific treatments were defined for each target and are available in Table 1.

**Table 1:** Description of targets and treatments with respective dosages according to the entomopathogenic microorganisms tested

Target	Treatment	Dosage (L ha^−1^)	Origin
*Spodoptera frugiperda* (corn earworm)	*Saccharopolyspora spinosa*	7.0	Bioinputs/macroscale
	*Bacillus thuringiensis*	8.0	Bioinputs/macroscale
	*Bacillus thuringiensis/Saccharopolyspora spinosa*	4.0/5.0	Bioinputs/macroscale
	*Bacillus thuringiensis*	0.8	Xentari/commercial
	Control	-	-
*Dalbulus maidis* (corn leafhopper)	*Saccharopolyspora spinosa*	7.0	Bioinputs/macroscale
	*Chromobacterium subtsugae*	7.0	Bioinputs/macroscale
	*Cordyceps fumosorosea* *On farm*^α^	7.0	Bioinputs/macroscale
	*Saccharopolyspora spinosa/C. subtsugae*	5.0/5.0	Bioinputs/macroscale
	*Beauveria bassiana*	7.0	Bioinputs/macroscale
	*Cordyceps fumosorosea*	0.8	Octane/commercial
	Control	-	-
*Ceratitis capitata* (fruit fly)	*Saccharopolyspora spinosa*	10 mL L^−1^ (among others)^β^	Bioinputs/macroscale
	Spinosyns J and L	5 mg L^−1β^	Spinetoram 25%/commercial
	Control	-	-

^α^Production in on-farm mode was carried out according to the procedure described in item 2.2 at a production plant located in a rural area: Takaoka Farm, Marília, São Paulo-SP (21°59′44.95″ S, 49°58′45.98″ W). ^β^Dose used in the bait for the purposes of the laboratory experiment

#### Bioassay with 2nd instar caterpillars of *Spodoptera frugiperda*

The fall armyworm, *S. frugiperda*, was acquired from a commercial supplier (Biopartner®). The insects were maintained on an artificial diet (similar to that used at the Luiz de Queiroz College of Agriculture – ESALQ/USP) in a climate-controlled room at 26 °C, with adults fed a honey solution.

To evaluate the efficiency of the bioinsecticides, the substrate, sorghum leaves, was immersed in the solution containing its respective active ingredient. The solutions containing the bioinsecticides were prepared according to item 2.4, using the equivalent of an application volume of 200 L ha^−1^. New, healthy leaves were collected, washed with distilled water to remove dirt and contaminants, and then dried on paper towels. The leaves were cut into 2 × 4 cm rectangles and immersed in the bioinsecticide solution for 5 s. The leaves were removed with tweezers and left to dry for 30 s on paper towels in a place with air flow. After drying, the leaves were transferred to plastic jars with lids.

A second instar caterpillar was placed in each jar. For each treatment, one caterpillar was used per repetition, totaling 40 repetitions per treatment. The caterpillars received leaves with inoculum only in the first feeding; in subsequent feedings, when necessary, leaves free of inoculum were provided. The jars were stored under controlled conditions of temperature 25 ± 2 °C, relative humidity 60 ± 2%, and photoperiod of 12 h. Insect mortality was assessed on the 1 st, 3rd, 7th, and 10th days after the experiment was set up.

#### Bioassay with nymphs and adults of *Dalbulus maidis*

The *D. maidis* specimens were acquired from the commercial supplier Biopartner®. The insects were maintained in cages on host plants in an open environment without temperature control.

To evaluate the efficiency of the treatments, a bioassay was performed with topical application on the substrate, bean leaves. The mixtures were prepared from the mixtures described in item 2.4 and sprayed onto the bean leaves with a CO_2_ backpack sprayer equipped with a fan-type nozzle, at a constant pressure of 40 psi, using a mixture volume also equivalent to 200 L ha^−1^.

Bean plants were used for the development of *D. maidis* nymphs and adults, as they were the basis of their diet. The insects (nymphs and adults) used for the tests were collected in the field and transferred to the pots, using a total of 7 nymphs and 7 adults of *D. maidis* per bean pot, with 5 replicates per treatment. Each pot was kept inside closed “cages” with voile. The pots were stored under controlled conditions of temperature 25 ± 2 °C, relative humidity 60 ± 2%, and photoperiod of 12 h. The insects were evaluated for mortality for 12 days after application. After the final evaluation, the dead insects were placed in a Petri dish on sterile cotton soaked in water, simulating a humid chamber, and conditioned in a BOD incubator for 12 h of photoperiod with UVA light at 25 °C for 5 days.

#### Bioassay with *Ceratitis capitata* adults

Adult *C. capitata* were obtained from the breeding stock maintained at the Biological Control Laboratory at the State University of Montes Claros, Janaúba-MG (25° ± 1 °C, 70 ± 10% RH, and 14 h of light). During the insect breeding process, the larvae were fed an artificial diet based on wheat bran, soybean meal, regular sugar, and brewer’s yeast. The adults were reared in wooden cages measuring 20 × 20 × 20 cm and fed until the fifth day of age with an artificial diet based on common sugar, brown sugar, protein nutritional supplement, and Biones (Tanaka et al. 1969).

For the bioassays, 5-day-old male and female *C. capitata* adults were removed from the breeding cage with the aid of a silicone suction cup and transferred to cages adapted from 500-mL plastic containers. Four pairs of fruit flies and a wad of hydrophilic cotton soaked in water were placed in each cage. These insects were subjected to a period of nutritional stress for 12 h. After this period, 40 µL of the toxic bait was offered on a 2.0 × 2.0 cm piece of PET plastic with the aid of a graduated pipette, according to the dosage established for each treatment.

Two experiments were conducted. In the first experiment, concentrations of 5 mg L^−1^ (a.i.), 10 mg L^−1^ (a.i.), 15 mg L^−1^ (a.i.), 20 mg L^−1^ (a.i.), and 25 mg L^−1^ (a.i.) of the commercial product Spinetoram (25%) were used. In the second experiment, the bioinput produced by *S. spinosa* was diluted in water at the following concentrations: 2 mL L^−1^, 4 mL L^−1^, 6 mL L^−1^, 8 mL L^−1^, and 10 mL L^−1^. The baits were composed of different concentrations of the bioinsecticide or commercial product mixed with 5.0% hydrolyzed protein, used as a food attractant for adult fruit flies. A glass rod was used to mix the solutions. The procedures with the insects and preparation of the baits for the two experiments were the same.

##### Determination of the minimum effective concentration for the control of *Ceratitis capitata*

To determine the minimum effective concentration, two separate experiments were conducted, both using the toxic bait method with a 5.0% hydrolyzed corn protein attractant. All bait solutions were prepared by thoroughly mixing the respective product, the food attractant, and water in a 500 mL beaker until 1 L of final solution was obtained. The prepared baits were then offered on 2.0 × 2.0 cm pieces of PET plastic using 40 µL per bait.

##### Commercial insecticide Spinetoram (25%) (first experiment)

The product was diluted to achieve five concentrations of active ingredient (a.i.), with the following final product concentrations: 20 mg L^−1^ (5 mg L^−1^ of a.i.), 40 mg L^−1^ (10 mg L^−1^ of a.i.), 60 mg L^−1^ (15 mg L^−1^ of a.i.), 80 mg L^−1^ (20 mg L^−1^ of a.i.), and 100 mg L^−1^ (25 mg L^−1^ of a.i.). For the control, only water and 5.0% hydrolyzed protein were used.

The variable evaluated was mortality at 5, 9, 20, 25, 30, 35, 44, 49, 54, 62, and 72 h after treatment (HAT).

##### Bioinsecticide produced by *Saccharopolyspora spinosa* (second experiment)

The bioinput was diluted in water at the following concentrations: 2 mg L^−1^, 4 mg L^−1^, 6 mg L^−1^, 8 mg L^−1^, and 10 mg L^−1^. The control consisted of water and food attractant, hydrolyzed protein at 5%. An additional treatment was included, where the flies were offered bait containing water and the concentration of Spinetoram (25%) determined in the first experiment and 5.0% hydrolyzed protein.

#### Data analysis

Mortality was assessed at the proposed intervals after exposure, with insects considered dead upon the total absence of movement or reaction to two to three gentle tactile stimuli (fine brush) to the legs, antennae, or abdomen. Death was confirmed by persistent immobility in subsequent observations and by visual signs such as darkening and loss of body turgor. To compensate for natural mortality, all data were adjusted using the formula described by Abbott (1925).

##### Statistical analysis of *Spodoptera frugiperda* and *Dalbulus maidis*

A completely randomized experimental design (CRD) was adopted, with 40 replicates for *S. frugiperda* and 5 replicates for *D. maidis* (calculated separately for nymphs and adults); the data on the number of dead/live insects were submitted to analysis of variance (ANOVA) by the *F*-test (Ferreira et al. 2014), and if significant, treatments were compared by Tukey’s test at 5% probability; finally, the control efficiency (%) of the treatments was determined based on the mortality corrected by the control, using Abbott’s formula (Abbott 1925), with the Sisvar software, version 5.8, used for statistical analysis.

##### Statistical analysis of *Ceratitis capitata*

The experiment was conducted in a completely randomized experimental design (CRD) and consisted of five treatments (baits with different concentrations of Spinetoram (25%) and a control (baits with water and food attractant). Each treatment consisted of eight replicates, totaling 48 experimental units (cages containing four fly pairs each) and 192 pairs evaluated. At each evaluation, the number of dead flies was counted and recorded. For this experiment, the mortality values of *C. capitata* adults were corrected using the Schneider-Orelli formula (Püntener and Zahner 1981). The data were subjected to analysis of variance. The standard errors of the means between the mortalities found were calculated.

The experiment was conducted in a completely randomized design (CRD) and consisted of six treatments: baits with different concentrations of the bioinput produced by *S. spinosa* and Spinetoram (25%) and the control, baits with water and food attractant. Each treatment consisted of eight replicates, for a total of 56 experimental units (cages containing four fly pairs each) and 224 insect pairs evaluated. The mortality variable analyzed was determined under the same conditions as in the first experiment. The results were transformed to √x to meet the premise of parametric statistics performed by Bartlett’s test for homoscedasticity of variances and Shapiro Wilk’s test for data normality, both at a 5% probability of error. Subsequently, they were subjected to analysis of variance, and the comparison between the treatment means was performed using the Scott Knott test, at a 5% probability. The statistical program used was Sisvar (Ferreira 2000). Linear regression analysis and the binomial Probit model (Flores and Gomez, 2011) were also performed, which were adjusted by the chi-square test to determine the median lethal time (LT_50_). The software used to perform this test was the Statistical Package for the Social Sciences (SPSS), version 2.0.

## Results

### Chemical composition of the bioinput produced by *Saccharopolyspora spinosa* as a function of shelf life

Through HPLC-MS analysis, it was possible to propose an identification of the chemical composition of the bioinputs produced by *S. spinosa* after 15, 90, and 180 days (Table 2).

**Table 2:** Analytical data of the metabolites found in the bioinput produced by *Saccharopolyspora spinosa* as a function of shelf life

Sample/shelf life (days)	Identification proposal	Retention time (*t*_R_) (min)	Ionization mode	Molecular formula	*m/z* experimental	*m/z* theoretical	Error (ppm)	Reference
90; 180	Valine	0.542	Positive	C_5_H_11_NO_2_	118.0858	118.0862	−3.39	Metabolomics Workbench
180	Glyceric acid	0.644	Negative	C_3_H_6_O_4_	151.0238 ^δ^	151.0248	−6.62	Metabolomics Workbench
15; 90; 180	Leucine	0.741	Positive	C_6_H_13_NO_2_	132.1015	132.1019	−3.03	Ye et al. (2020)
15; 90; 180	Phenylalanine	1.407	Positive	C_9_H_11_NO_2_	166.0855	166.0878	−4.21	Sun et al. (2014)
15	Tryptophan	2.390	Positive	C_11_H_12_N_2_O_2_	205.0977	205.0972	2.44	Basumatary et al. (2023)
180	Unknown I	2.876	Negative	–	181.0444	–	–	–
15	Cyclo(Met-Val)	3.022	Positive	C_10_H_18_N_2_O_2_S	231.1150	231.1162	−5.19	Metabolomics Workbench
15; 90	N-stearoyl tryptophan	3.572	Positive	C_29_H_46_N_2_O_3_	453.3463	453.3475	−2.64	Metabolomics Workbench
15	Sphinganine	4.038	Positive	C_18_H_39_NO_2_	340.2618^γ^	340.2612	1.76	HMDB
15	Unknown II	4.221	Positive	–	396.8039	–	–	–
90; 180	3-Hydroxyhexanoic acid	5.000	Negative	C_6_H_12_O_3_	131.0715	131.0714	0.76	Metabolomics Workbench
15	Spinosyn A	6.502	Positive	C_41_H_65_NO_10_	732.4694	732.4681	1.77	Rahman et al. (2021)
90	2-[(E)−5-Methyl-3-oxododec-4-enimidoyl]oxyhexa-noic acid	6.166	Negative	C_19_H_33_NO_4_	384.2411^δ^	384.2392	4.94	Metabolomics Workbench
15	Spinosyn D	6.735	Positive	C_42_H_67_NO_10_	746.4833	746.4838	−0.67	Rahman et al. (2021)
180	Unknown III	7.039	Negative	–	204.0602	–	–	Metabolomics Workbench
15	Penicitrinol D	8.283	Positive	C_16_H_22_O_4_	279.1599	279.1591	2.86	Chen et al. (2011)
90; 180	PG(O-18:0/0:0)	8.447	Negative	C_24_H_51_O_8_P	497.3246	497.3249	−0.60	Metabolomics Workbench
	Phosphattidylglyce-rols							
90; 180	Cyclo-(L-Ile-L-Leu-L-Leu-L-Leu-L-Leu)	9.784	Positive	C_30_H_55_N_5_O_5_	566.4270	566.4276	−1.06	Metabolomics Workbench
90; 180	PE(O-20:0/4:0)	10.212	Negative	C_29_H_60_NO_7_P	610.4077^δ^	610.4090	−2.13	Metabolomics Workbench
	Phosphatidyletha-nolamines							
15	Diethylhexyladipate	11.313	Positive	C_22_H_42_O_4_	371.3155	371.3156	−0.27	Gotthardt et al. (2019)
90; 180	PA(18:0/20:4)	11.395	Negative	C_41_H_73_O_8_P	723.4899	723.4970	−7.10	Wang et al. (2016)
	Phosphtatidic acids							
90; 180	PC(P-18:1(9Z)/20:4(5Z,8Z,11Z,14Z))	11.849	Positive	C_46_H_82_NO_7_P	814.5768^ε^	814.5721	5.77	HMDB
	Phosphatidylcho-lines							
90; 180	PS (16:0/20:0)	12.4	Negative	C_42_H_82_NO_10_P	836.5683	836.5658	2.99	Metabolomics Workbench
	Phosphattidylserines							
90; 180	Unknown IV	13.193	–	–	949.6493	–	–	–

^γ^[M + K]^+^; ^δ^[M + HCOOH]^–^; ^ε^[M + Na]^+^. HMDB: https://www.hmdb.ca/. Metabolomics Workbench: https://www.metabolomicsworkbench.org/databases/metabolitedatabase.php

### Quantification of spinosyns A and D in the bioinput produced by *Saccharopolyspora spinosa*

**Fig. 1:**
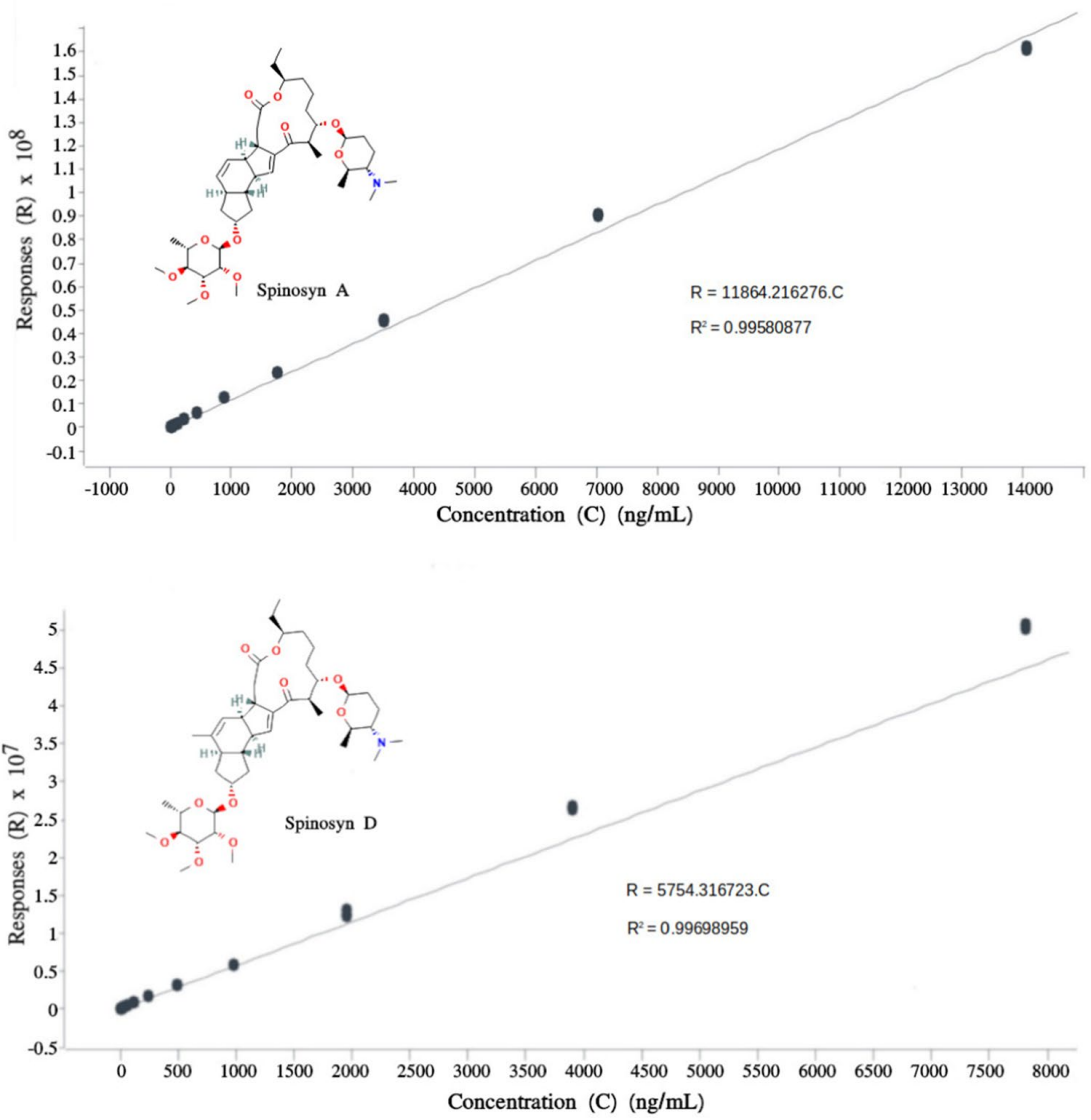
Calibration curves of spinosyns A and D. The insert contains the equation obtained, the error in the form of a linear correlation coefficient, and the structural formulas of analogs A and D, respectively (https://pubchem.ncbi.nlm.nih.gov/compound/Spinosyn-A, https://pubchem.ncbi.nlm.nih.gov/compound/Spinosyn-D)

Figure 1 shows the calibration curves obtained in absolute response, equivalent to the area of the chromatograms obtained for spinosyns A and D, respectively (results not shown).

### Bioassay with 2nd instar caterpillars of *Spodoptera frugiperda*

The mortality rate of 2nd instar *S. frugiperda* and the control efficiency according to the entomopathogenic microorganisms tested are available in Table 3.

**Table 3:** Mortality of 2nd instar *Spodoptera frugiperda* caterpillars fed on corn leaves soaked with different bioinsecticides

Treatments	1 DAA			3 DAA			7 DAA			10 DAA		
	ND (unit)	MO (%)	CE (%)	ND (unit)	MO (%)	CE (%)	ND (unit)	MO (%)	CE (%)	ND (unit)	MO (%)	CE (%)
*Saccharopolyspora spinosa*	0	0.0 b	0.0	8	20.0 b	20.0	28	70.0 b	67.6	33	82.5 a b	80.6
*Bacillus thuringiensis*	0	0.0 b	0.0	1	2.5 b	2.5	16	40.0 c	35.1	22	55.0 c	50.0
*B. thuringiensis/S. spinosa*	0	0.0 b	0.0	8	20.0 b	20.0	20	50.0 b c	45.9	26	65 b c	61.1
*B. thuringiensis (Xentari)*	31	77.5 a	77.5	31	77.5 a	77.5	39	97.5 a	97.3	39	97.5 a	97.2
Control	0	0.0 b	-	0	0.0 b	-	3	7.5 d	-	4	10.0 d	-
Coefficient of variation/average	7.36/15.5			12.29/24.0			13.71/53.0			12.69/62.0		
Standard deviation/standard error	0.45/0.20			0.40/0.18			0.33/0.15			0.32/0.14		

Means followed by the same letter do not differ from each other by the Tukey test (*p* < 0.05); data was transformed into √*x* + 1; total insects per treatment: 50; average insects per treatment: 5

*DAA*, days after application; *ND*, number of deaths; *MO*, mortality; *CE*, control efficiency; *CV*, coefficient of variation

### Bioassay with nymphs and adults of *Dalbulus maidis*

Data on mortality of *Dalbulus maidis* nymphs and adults and control efficiency with different entomopathogenic microorganisms are available in Table 4.

**Table 4:** Mortality rate of *Dalbulus maidis* and control efficiency according to the entomopathogenic microorganisms tested

Treatments	12 DAA						17 DAA	
	DN (unit)	CE (%)	DA (unit)	CE (%)	TD (unit)	CE (%)	PDN (%)	PDA^NS^ (%)
*Chromobacterium subtsugae*	1.6 c d	18.2	0.4 b	2.9	2.0 b c	10.4	0.0 c	0.0
*Cordyceps fumosorosea*	4.6 a b	63.6	1.8 a b	23.5	6.4 a	43.3	96.0 a	37.7
*S. spinosa/C. subtsugae*	2.8 b c	36.4	1.8 a b	23.5	4.6 a b	29.9	0.0 c	0,0
*Beauveria bassiana*	4.2 a b	57.6	2.8 a	38.2	7.0 a	47.8	40.0 b c	45.0
*Cordyceps fumosorosea (On farm)*	4.6 a b	63.6	2.6 a	35.3	7.2 a	49.3	96.0 a	18.3
*S. spinosa*	3.4 a b c	45.5	1.0 a b	11.8	3.4 a b	28.4	100.0 a	15.3
*Cordyceps fumosorosea* (Octane)	5.2 a	72.7	1.6 a b	20.6	6.8 a	46.3	80.7 a b	26.3
Control	0.4 d	-	0.2 b	-	0.6 c	-	0.0 c	0.0
Coefficient of variation/average	31.84/3.35	-	66.39/1.52	-	36.69/4.87	-	40.91/51.58	159.47/17.83
Standard deviation/standard error	1.66/0.59	-	0.94/0.33	-	2.47/0.87	-	46.68/16.50	17.59/6.22

Means followed by the same letter do not differ from each other by the Tukey test (*p* < 0.05);  *NS*, not significant by Tukey’s test (*p* < 0.05); total insects per repetition: 14 (7 nymphs and 7 adults)

*DAA*, days after application; *DN*, dead nymphs; *CE*, control efficiency; *TD*, total deaths; *DA*, dead adults; *PDN*, proven death of nymphs; *PDA*, proven death of adults; *CV*, coefficient of variation

### Bioassay with *Ceratitis capitata* adults

The determination of the minimum effective concentration of the commercial insecticide Spinetoram (25%) for the control of *Ceratitis capitata* was investigated. There was no significant difference (*p* = 0.1871) in the mortality rates of *C. capitata* adults after 72 h (HAT) of ingestion of the toxic baits (Table 5).

Table 6 shows the mortality of *Ceratitis capitata* after ingestion of toxic baits based on the multiplication of *Saccharopolyspora spinosa* after 72 h (HAT).

The lethal times LT_50_ and LT_60_ at a concentration of 10 mL L^−1^ of bioinput produced by *S. spinosa* to reduce 50.0% and 60.0% of *C. capitata* populations are available in Table 7.

### Economic feasibility study of *in vitro* experimental treatments aiming at their scaling and use in field management

Table 8 shows the current cost in dollars of each product used in treatments to control *S. frugiperda*, *D. maidis*, and *C. capitata* according to the dosage used to obtain the management cost per hectare.

## Discussion

### Chemical composition and quantification of spinosyns A and D

Table 2 shows the chemical composition of the High Tech bioinputs produced by *S. spinosa*, revealing the complexity and multifunctionality of these inputs due to the significant diversity of bioactive compounds with potential applications in agriculture.

A total of 26 compounds were presented, as shown in the mass spectra available in the Supplementary Material. The identification proposal was made considering an error of less than 10 ppm. Some compounds with significant signals in the spectrum were not identified in the literature and databases available. There are four such compounds, which are listed below in descending order of polarity: Unknown I (*t*_R_ = 2.876; *m/z* = 181.0444), Unknown II (*t*_R_ = 4.221; *m/z* = 396.8039), Unknown III (*t*_R_ = 7.039; *m/z* = 204.0602), and Unknown IV (*t*_R_ = 13.193; *m/z* = 949.6493).

Among the 10 main compounds identified in the sample with a shelf life of 15 days, we found two essential amino acids: leucine and phenylalanine, with *m/z* 132.1015 and 166.0855, respectively. Leucine is the compound with the highest polarity, with a retention time of 0.741 min, and although it is an essential amino acid for many bacteria, including some of the genus *Saccharopolyspora*, its role in the biosynthesis processes of *S. spinosa* for the production of metabolites, as has already been elucidated for glutamate and aspartate (Zhang et al. 2021), both of which are indispensable amino acids in the biosynthesis of spinosyns.

Tryptophan also has high relative polarity and was identified at a *t*_R_ of 2.390 min with *m/z* 205.0977. Tryptophan does not participate directly in the biosynthesis of spinosyns, but *S. spinosa* can convert it into secondary metabolites, such as *N*-stearoyl tryptophan, which was also identified at a *t*_R_ of 3.572 min and *m/z* 453.3463. To the best of our knowledge, there are no reports in the literature on its effects on pest control; however, some analogous metabolites, derived from fatty acids and amides produced by *Streptomyces* species, have shown significant insecticidal activity against *Helicoverpa armigera* (Hübner) larvae. Mortality rates ranged from 70 to 78% in laboratory, greenhouse, and diet impregnation trials, with LD₅₀ and LD₉₀ values of 627 ppm and 2276 ppm, respectively, indicating considerable insecticidal potency at these concentrations (Gopalakrishnan et al. 2016).

The cyclic dipeptide Cyclo(Met-Val) was identified at a *t*_R_ of 3.022 min and belongs to the class of compounds called diketopiperazines (DKPs), which have shown promising activities for biological pest control. Cyclo(Pro-Val), for example, was isolated from the entomopathogenic fungus *Nomuraea rileyi* and showed insecticidal activity against *Oncopeltus fasciatus* (Dallas) (Marcinkevicius et al. 2017). Other DKPs such as Cyclo(Pro-Leu) and Cyclo(Pro-Phe), also derived from *N. rileyi*, showed insecticidal activities. Cyclo(Pro-Val) produced by *Bacillus velezensis* demonstrated strong antifungal activity against *Colletotrichum gloeosporioides*, a pathogen responsible for anthracnose in plants, highlighting its potential as a bioprotection agent (Choub et al. 2021).

Sphinganine, identified at a *t*_R_ of 4.038 min and *m/z* 340.2618, was associated with cell death in plants such as *Arabidopsis thaliana* in response to insect eggs. Studies indicate that sphinganine may be involved in signaling pathways that mediate long-chain base-induced cell death in plants, suggesting a potential role in defense against herbivores (Markham et al. 2011; Saucedo-García et al. 2011; Schütze, 2018).

Spinosyns A and D are among the compounds of intermediate polarity found, with *t*_R_ of 6.502 and 6.735 min, and *m/z* 732.4694 and *m/z* 746.4833, respectively. Spinosyns are chiral molecules containing multiple stereogenic centers in their structure, which includes a system of fused rings, sugar groups, and pyran units, as well as functional groups such as amines and esters (see insert in Fig. 1). The main structural difference between spinosyns A and D lies in the configuration of the C-23 carbon atom: in spinosyn A, this carbon has the (*R*) configuration, while in spinosyn D, the configuration is (*S*), although they are not enantiomers due to a structural factor. Spinosyn D (C₄₂H₆₇NO₁₀) has a methyl (-CH₃) substituent on the 6-membered ring with an unsaturation located in the central part of the molecule, while spinosyn A (C₄₁H₆₅NO₁₀) has no substituent (-H) on that same ring (Rahman et al. 2021). This can be confirmed exactly by HPLC-MS testing, with a mass difference equivalent to 14.0122 u (-CH₂), as shown in Table 2. The replacement of a hydrogen by a methyl group makes spinosyn D slightly more nonpolar than spinosyn A, which was corroborated in this study by the longer retention time (+ 0.233 min). Spinosyns were not identified in samples with a shelf life of 90 and 180 days, indicating that they are not stable during prolonged storage periods.

A new dimer and a new monomer of citrinin, named penicitrinol A and penicitrinol L, respectively, were identified by HPLC and isolated from the co-culture of *A. sydowii* EN-534 and *P. citrinum* EN-535 (Yang et al. 2018). Penicitrinols A and B are derivatives of citrinin and can be produced by *Penicillium* sp. H9318, a filamentous soil fungus (Yao et al. 2011). Mangroves are highly productive ecosystems that harbor a diverse range of fungal species, and penicitrinols J and K have been identified in this environment (Braga et al. 2024). Penicitrinol D was identified in the bioinput produced by *S. spinosa*, the second most apolar of all compounds found in the sample with a shelf life of 15 days, with a *t*_R_ of 8.283 min and a *m/z* 279.1615. This is an uncommon analogue, and it is important to note that this class of compounds is a potential antimicrobial agent.

The bioinput samples produced by *S. spinosa* with a shelf life of 90 and 180 days presented 15 compounds, among them 3 already identified in the 15-day samples; therefore, they are quite stable, such as leucine, phenylalanine, and *N*-stearoyl tryptophan. Thus, 15 new compounds emerged, indicating that the bioinput continues to undergo important chemical changes during storage or after application in the field. In fact, less stable molecules, such as spinosyns, degrade, and others are formed that were only detected after more than 90 days of storage. Among the main compounds identified in samples with a shelf life of 90 and 180 days are amino acids, phospholipids, complex fatty acids, and other secondary metabolites.

Valine, a highly polar amino acid identified at a retention time *t*_R_ of 0.542 min and *m/z* 118.0858, is strategically important because it acts as a precursor in bioactive compounds with insecticidal activity, such as fluvalinate, a synthetic pyrethroid used to control various agricultural pests. In addition, derivatives such as valine menthyl ester (ment-Val) demonstrate the ability to induce defenses in plants against herbivores such as *Spodoptera litura* (Fabricius) (Sarkar et al. 2021).

Glyceric acid, another highly polar compound, was identified at a *t*_R_ of 0.644 min and a *m/z* 151.0238. Its molecule contains three carbons involved in secondary metabolic pathways in plants, particularly in the photorespiration cycle. Although it has no known insecticidal activity, it is associated with plant resilience, contributing to the response to abiotic stresses and cellular redox balance (Timm et al. 2012).

Phosphatidylglycerol (PG), identified at a *t*_R_ of 8.447 min and a *m/z* 497.3546, is an essential phospholipid in plant membranes, especially in chloroplast thylakoids, where it participates in the structure of the photosynthetic complex, cell signaling, and response to abiotic stress (Wada and Murata 2009).

The cyclic pentapeptide Cyclo-(L-Ile-L-Leu-L-Leu-L-Leu-L-Leu), with a *t*_R_ of 9.784 min and a *m/z* 566.4270, is rich in hydrophobic amino acids, providing structural stability and resistance to degradation. Normally produced by *Bacillus*, it has antifungal and antibacterial activity, acting on the permeabilization of pathogen membranes (Ongena and Jacques 2008).

Phosphatidylethanolamines (PEs), detected at a *t*_R_ of 10.212 min with a *m/z* 610.4077, especially variants such as PE(O-20:0/4:0), are precursors of signaling lipids, activating immune defenses and reinforcing resistance against pathogens (Wang et al. 2020).

Di(acylalkyl)glycerols (DAGs), with a tR of 11.016 min and a *m*/*z* 701.4291, are important precursors in the biosynthesis of phospholipids, such as phosphatidylcholine and phosphatidylethanolamine, and are also involved in cell signaling, especially during responses to biotic and abiotic stresses (Block et al. 2007).

Phosphatidic acid PA(18:0/20:4), detected at a *t*_R_ of 11.395 min and with a *m/z* 723.4899, is a signaling lipid that activates defense genes, reactive oxygen species (ROS), and cell wall strengthening. Although not a direct insecticide, it contributes to resistance against pests such as caterpillars and flies (Okazaki and Saito 2018).

Phosphatidylserine (PS) (16:0/20:0), the most apolar compound identified, showed a *t*_R_ of 12.400 min and a *m/z* 836.5683. It participates in membrane formation and cell signaling. When exposed to apoptotic cells, it induces an anti-inflammatory response in macrophages and plays a role in the infectious process of parasitic protozoa (Rodrigues et al. 2018).

As can be seen, the chemical composition of the bioinput produced by *S. spinosa* reveals the presence of compounds with proven activity, such as spinosyns A and D, widely recognized for their direct insecticidal action against various agricultural pests. In addition, compounds with indirect or defensive insecticidal potential have been identified, such as sphingolipids and fatty acid derivatives, which can activate defense mechanisms in plants or interfere with insect physiology.

The bioinput produced by *S. spinosa* also contains compounds with complementary and multifunctional action, including metabolites with antimicrobial, cytotoxic, and cell signaling properties, broadening its spectrum of action. The chemical diversity observed also suggests probable synergism potential and a multi-target effect, which may result in greater effectiveness in pest control and a lower risk of resistance to crops. Studies have shown that the *Spodoptera exigua* (Hübner) caterpillar and the *Culex quinquefasciatus* Say mosquito are resistant to the specific use of pure spinosyns found in the products Spinosad and Spinetoram (Zuo et al. 2020; 2022; Hafez and Abbas 2021).

Therefore, this bioinput is capable of aligning biological efficiency, production scalability, and environmental sustainability, making it a sustainable and promising alternative in integrated pest management, among others, such as the corn earworm, corn leafhopper, and fruit fly, which are highly destructive agricultural pests causing severe economic losses in various crops. They cause direct damage by feeding on plant tissues and indirect damage by transmitting diseases, compromising crop productivity and quality. Their high adaptability, rapid reproduction, and resistance to insecticides make control particularly challenging. These pests mainly affect tropical and subtropical regions of the Americas, Africa, southern Europe, and Asia, where large areas of corn, fruit, and vegetable production are concentrated (Díaz et al. 2024).

In the bioinput samples analyzed with approximately 15 days of multiplication of *S. spinosa* and using the equations shown in Fig. 1, 29.4 ± 3.6 and 13.3 ± 1.5 mg L^−1^ of spinosyns A and D were found, respectively, totaling 42.7 mg L^−1^ of total spinosyns. The LOQ and LOD values obtained were 13.7 ng L^−1^ and 4.16 ng L^−1^ for spinosyn A and 7.63 ng L^−1^ and 2.31 ng L^−1^ for spinosyn D, respectively.

Although this value is lower than those commonly reported in studies > 200 mg L^−1^ (Jin et al. 2009; Jha et al. 2014), it is worth noting that these results were obtained in an 800-L reactor, that is, on an industrial scale, which highlights the robustness and applicability of the methodology, unlike other studies obtained in reactors normally with a capacity < 5 L. This information is important, as the high yields published in the literature often depend on highly optimized conditions, specific genetic modifications, and expensive substrates (Madduri et al. 2001a, b; Yang et al. 2016), which are economically unfeasible on a large scale.

The concentration of approximately 43 mg L^−1^ is equivalent in mass to almost 34.16 g of total spinosyns in an 800-L reactor. In the experiments conducted here, as well as in agricultural practices, the dosage of 7 L ha^−1^ of this bioinput produced by *S. spinosa* is commonly used, as described in Table 1. In addition, the procedure adopted in agricultural practice normally involves a dilution in water whose volume varies depending on the target pest, level of infestation, crop cultivated, and form of application. For the control of *C. capitata* in fruit growing and considering medium-sized plant canopies, the final spray volume varies between 200 and 400 L, promoting a dilution factor in which the final concentration of spinosyns is between 0.75 and 1.5 mg L⁻^1^.

However, for citrus farming, a higher dilution factor is adopted and the final spray volume under these conditions can range from 1500 to 2000 L ha^−1^, in which the total spinosyn concentration varies between 0.1 and 0.3 mg L^−1^. Studies have shown that spinosyns, even at these very low concentrations, such as those found in this study, are already technically effective in controlling several pests, such as 0.27 mg L⁻^1^ for *P. xylostella*, 1.23 mg L⁻^1^ for aphids, and 1.92 mg L⁻^1^ for *T. palmi* (Xu et al. 2024).

Control of *S. frugiperda*, which is currently considered one of the most widespread pests worldwide, requires higher concentrations in agricultural practice, where the LC_50_ normally ranges from 5 to 10 mg L⁻^1^. For this purpose, in areas considered smaller and difficult to access, drones are usually used with spraying solutions, where the final volume is smaller, ranging from 15 to 30 L.

Pest control can usually be carried out with insecticides and/or (bio)insecticides, which, in addition to drones, use airplanes or tractors. In the specific case of *D. maidis*, the dosages are 2 to 4 L ha^−1^, 2 to 5 L ha^−1^, and 4 to 7 L ha^−1^, with spray volumes ranging from 8 to 20 L, 10 to 30 L, and 25 to 100 L, respectively, with a final concentration of total spinosyn between 1.5 and 10.0 mg L^−1^.

### *In vitro* bioinsecticide efficiency

#### *Spodoptera frugiperda*

Table 3 shows the results obtained that present significant differences in the efficacy of entomopathogenic microorganisms used to control *S. frugiperda*. The formulation of (Xentari) based on *Bacillus thuringiensis* (Bt) stood out for presenting 77.5% mortality on the first day after application, which is not typical of bioinsecticides that depend exclusively on ingestion for lethal action. This early mortality may be related to a high cellular concentration of Bt in the commercial product and/or the presence of adjuvants in the formulation, which, under controlled microclimate conditions, could promote physiological stress in insects through unconventional pathways, such as respiratory interference or effects on cuticular integrity. From the third day after application, the entomopathogenic action of the other treatments began to be evident. The experimental isolate of *S. spinosa* showed 20% mortality, a performance equivalent to the combination of *B. thuringiensis*/*S. spinosa*, suggesting a lack of synergism between these agents in this initial phase of control. On the other hand, bioinputs produced on macroscale by *B. thuringiensis* resulted in only 2.5% mortality, revealing low efficacy under the conditions tested.

After the 7-day period of application, the control with *S. spinosa* showed significant improvement, reaching 70% mortality, being the second most efficient treatment after the positive control (Xentari), which reached 97.5%. This result demonstrates the progressive entomopathogenic potential of *S. spinosa* over time. The control with Bt produced on macroscale and isolated showed late improvement, with 55% mortality 10 days after application, being lower than the treatment with *S. spinosa* and the combination of both, which reached 82.5% and 65.0% mortality, respectively.

The lack of synergistic effect between *B. thuringiensis* and *S. spinosa* can be attributed to the difference in modes of action, while the former depends on the ingestion and release of δ-endotoxins in the mesentery wall; the latter acts through spinosyns, which affect the central nervous system by activating nicotinic acetylcholine receptors (Bravo et al. 2020).

Overall, the data obtained indicate that the *S. spinosa* isolate evaluated has promising potential for the development of a new bioinput aimed at controlling *S. frugiperda*, standing out for its consistent performance over time and for its pilot-scale production of 800 L. This result is relevant, especially when compared to most studies in the literature, in which effective concentrations of spinosyns are achieved only in benchtop reactors, with significantly smaller volumes (Madduri et al. 2001a, 2001b; Yang et al. 2016).

Such evidence highlights the technical advance in intermediate-scale production and reinforces the biotechnological value of the isolate tested. Furthermore, the results demonstrate that the bioinput produced by *S. spinosa*, after 10 days of application, already exerts a statistically equivalent effect to that of the commercial product based on *B. thuringiensis* (Xentari) on *S. frugiperda* caterpillars.

#### *Dalbulus maidis*

In the pre-test, all treatments had an initial number of 7 nymphs and 7 adults. The results shown in Table 4 show mortality with significant variations between both treatments throughout the evaluations.

After 12 days of application, the treatment with the bioinput produced by *S. spinosa* presented a mortality of 45.5%, while the commercial product, Octane, showed the highest mortality rate for nymphs, reaching 72.7% at this stage. The mortality for adults in this period is 11.8% and 20.6% using the *S. spinosa* and Octane treatments, respectively, demonstrating that the treatment using the commercial product is almost twice as efficient at this stage.

The opposite is observed after 17 days of application for nymphs, in which the bioinput produced by *S. spinosa* presented an extraordinary result of 100.0% mortality against 80.7% for the commercial product, Octane, although statistically they are equivalent. This indicates a high efficacy of the bioinputs produced by *S. spinosa* in the infection and direct mortality of nymphs, suggesting that these microorganisms have an excellent capacity for penetration and colonization in the juvenile stage of the leafhopper. Observing the data for adults after 17 days of application, it can be seen that mortality using the bioinput produced by *S. spinosa* presented a result of 15.3% mortality compared to 26.3% for the commercial product, Octane.

The mortality rates for adults in both treatments were generally lower compared to nymphs, suggesting that adults may be more resistant to the treatments applied because they have a more resistant cuticle, being thicker and sclerotized, which may offer greater resistance to the penetration of fungal spores, and also because of greater movement, since adults have greater mobility and dispersion capacity, which may reduce exposure to the fungus and decrease the effectiveness of control.

On the other hand, the greater efficiency on nymphs may be attributed to their tendency to be more susceptible to infection by entomopathogenic fungi, since the cuticle of nymphs is generally thinner and less sclerotized compared to adults, facilitating the penetration of fungal spores. Another likely cause is that nymphs are often found in places with a more humid and protected microclimate (for example, at the base of plants or under leaves), which may favor the development and action of entomopathogenic fungi. They also have less movement capacity compared to adults, which may increase the exposure and effectiveness of fungi applied in the field.

The evaluation of the effectiveness of *Chromobacterium subtsugae* and *Beauveria bassiana* produced on macroscale for the control of *D. maidis* revealed contrasting performances between the two microorganisms. The treatment with *B. bassiana* demonstrated significantly superior performance in all parameters evaluated, while *C. subtsugae* showed low efficacy in the bioassay conducted, although both can be considered very poor for the target *D. maidis*. The data obtained show that both treatments with *Cordyceps fumosorosea*, both the one produced macroscale in industry and *on farm*, showed good efficacy in controlling *D. maidis*, with very similar performance in terms of total mortality and control efficiency, demonstrating the ease of replicating the biotechnological procedure in loco close to the application points.

#### *Ceratitis capitata*

Similar studies with spinosyns observed 100.0% mortality of *A. fraterculus* when ingesting toxic baits at a dose of 3.7 mg L^−1^ (Baldin 2018). Given the results obtained in the present study, the minimum concentration for controlling *C. capitata* was established as 5 mg L^−1^ of active ingredient, see Table 5. This concentration was also evaluated in the next experiment, together with those consisting of different concentrations of the bioinput produced by *S. spinosa*.

**Table 5:** Mortality of *Ceratitis capitata* adults after 72 h of ingestion of different doses of the synthetic insecticide Spinetoram (25%) offered to insects in toxic baits

Concentration (mg L^−1^)	Mortality^ζ^ (% ± (SE))
5	91.07 ± 4.04
10	98.22 ± 1.56
15	100.00 ± 0.00
20	100.00 ± 0.00
25	100.00 ± 0.00

^**ζ**^Mortality corrected by the Schneider-Orelli formula (Abbott 1925) e *SE*, standard error of the mean

The results available in Table 6 show that there was a statistical difference between the treatments in the mortality of *C. capitata* after ingestion of toxic baits based on the bioinput produced by *S. spinosa*, with emphasis on the treatment at a concentration of 10 mg L^−1^, whose mortality was higher than the other concentrations tested. When comparing the treatments of the multiplied product with the controls, it was observed that Spinetoram at a concentration of 5 mg L^−1^ caused a mortality of approximately 92%, differing statistically from all treatments, as well as from the control (water and hydrolyzed protein at 5.0%) (Table 6). This higher mortality of Spinetoram at a concentration of 5 mg L^−1^ may be related to the higher concentration of active ingredient present in the commercial product.

**Table 6:** Mortality of *Ceratitis capitata* adults after 72 h of ingesting toxic baits based on the bioinput produced by *Saccharopolyspora spinosa*

Treatments	Concentration (mL L^−1^)	Mortality^θ^ (% ± (SE))
*S. spinosa*	2	37.5 ± 4.72^c^
*S. spinosa*	4	39.06 ± 6.44^c^
*S. spinosa*	6	42.18 ± 4.69^c^
*S. spinosa*	8	50.00 ± 3.34^c^
*S. spinosa*	10	64.06 ± 4.38^b^
Spinetoram^ι^	5^η^	92.18 ± 4.05^a^
Control	-	12.5 ± 3.34^d^
CV (%)		26.58

^η^Spinetoram 5 mg L^−1^. ^θ^Means followed by the same letters in the column do not differ from each other by the Scott-Knott test, at the 5% probability level. ^ι^Water and 5% hydrolyzed protein. Data were transformed to √x. *SE*, standard error of the mean

The mortality rate of *C. capitata* after ingesting toxic baits based on bioinput produced through multiplication of the bacterium *S. spinosa* (results not shown) demonstrated that only 5 h after setting up the experiment, the occurrence of fly mortality reached values between 1.6 and 4.7% at concentrations of 4 mL L^−1^ and 10 mL L^−1^, respectively.

The highest mortality rate of flies was observed at the 10 mL L^−1^ bioinput concentration, which increased throughout the evaluations, reaching its maximum of 64.1% after 60 h; that is, 2.5 days after the experiment was set up (results not shown). For Spinetoram at a concentration of 5 mg L^−1^, the highest mortality rate of flies was observed around 30 h after the start of the evaluations. As observed in another study, due to the need to ingest the toxic bait, it is common for there to be a lower initial mortality rate and a gradual increase in mortality over time as a result of ingesting the insecticide (Baldin 2018).

After 9 h, Spinetoram showed a marked mortality with 12.5 to 32.8% of dead flies, and at this time, an increase in the number of flies was also observed in the control performed with bioinput produced by *S. spinosa*. At this time of monitoring, it was already possible to confirm that the synthetic product was the most toxic to the flies when comparing the different concentrations of bioinput produced by *S. spinosa*. Ingestion of toxic baits containing the synthetic insecticide Spinetoram caused a mortality rate ranging from 12.5 to 32.8% in the first 9 h of the experiment, compared to 1.3 to 4.1% for SM concentrations.

When comparing the mortality rates of adult flies that occurred after ingesting baits containing different concentrations of the biopesticide produced by *S. spinosa*, it was found that they were higher, approximately three times higher than those observed in the control, even at the lowest concentration of biopesticide, namely, 2 mg L^−1^. This fact suggests that, if higher concentrations of the metabolites produced by *S. spinosa* had been used, for example, higher than 10 mL L^−1^, the mortality values of *C. capitata* could have been much higher and equivalent to those found for Spinetoram, as observed in a linear regression, whose equation of the line: mortality (%) = 3.2031 × concentration (mL L^−1^) + 27.344 and *R*^2^ = 0.8629 showed (results not shown). Additionally, considering that the concentrations of bioinput normally used in the field are in the order of 0.5 to 1.0% (v v^−1^), which are equivalent to 5 mL L^−1^ and 10 mL L^−1^, respectively, this study establishes the need for reconsideration for field application. It is therefore suggested that the concentration of the bioinput produced by *S. spinosa* to control *C. capitata* populations be increased to ensure efficient control of the fruit fly, both in the form of toxic baits and by application to the entire area.

Even so, the bioinput produced by *S. spinosa* was effective in controlling *C. capitata* adults, considering the results obtained for the concentration of 10 mL L^−1^, where a mortality rate of almost 65% was obtained after 60 h of ingestion of the toxic bait by the respective insects. This result can be considered promising because, although the use of products such as Spinetoram or Spinosad is recommended in cases of greater fruit fly infestation pressure, the bioinput produced by *S. spinosa* may enhance fruit fly control or even reduce chemical dosages if used together or even to vary the active ingredients. In addition, a reduction in the persistence of these synthetic molecules in the field may be achieved with this new proposal for fruit fly control since, when it comes to isolated products, the persistence of toxic attractants based on Spinosad at 0.01% in fruit fly mortality caused significant mortality over a long period of 21 days (Borges et al. 2015). Naturally, a bioinput produced by *S. spinosa* in liquid medium would not bring the same persistence due to the lack of stabilization of the active ingredients by other molecules that were found in its formulation (Table 2), although it presupposes more applications for effective improvement of control.

The mortality of fruit flies in the control at the end of the experiment was 12.50%, a value considered low when compared to the other treatments (Table 6). The deaths in the control probably occurred due to the handling of the insects at the time of their transfer to the experimental cages, which was done using silicone suction cups and not with CO_2_, the method most commonly used for this type of procedure in the laboratory (Nunes et al. 2019).

The lethal times LT_50_ and LT_60_ at a concentration of 10 mL L^−1^ of bioinput produced by *S. spinosa* to reduce 50.0% and 60.0% of *C. capitata* populations were approximately 2 days (48.7 h) and 2.7 days (63.93 h), respectively (Table 7).

**Table 7:** Lethal time (LT) in hours (h) for *Ceratitis capitata* after ingestion of toxic baits contaminated with bioinput produced by *Saccharopolyspora spinosa* and for the commercial insecticide Spinetoram (25%)

Treatments	*N*	Inclination (± SE)	LT_50_ (IC95)^κ^	LT_60_ (IC95)^κ^	LT_90_ (IC95)^κ^	*χ*^2^	DF	*p*-value
Bioinput *S. spinosa* (10 mL L^−1^)	64	2.15 ± 0.22	48.7 b	63.93 b	-	6.31	6	0.39
Spinetoram (5 mg L^−1^)	64	2.85 ± 0.21	11.53 a	14.15 a	32.46	9.34	6	0.40

*N*, number of insects used; *CI*, confidence interval; *χ*^*2*^, chi-square; *DF*, degrees of freedom

^κ^Significant difference based on the 95% confidence intervals

These lethal times were longer than those observed for fruit flies that fed on toxic baits based on Spinetoram at a concentration of 5 mg L^−1^, TL_50_ equivalent to 11.53 h. These results are very similar to those found in another study conducted by Schutze and collaborators who evaluated the toxicity and residual effects of toxic baits based on spinosyns on the South American fruit fly, *A. fraterculus*, whose TL_50_ was equivalent to 11.43 h (Schütze et al. 2018).

### Environmental safety and toxicology of formulations containing spinosyns A and D

The environmental and toxicological safety of the active ingredient Spinosad, which comprises spinosyns A and D present in this bioinput, is widely established by international regulatory agencies such as the World Health Organization (WHO) and the US Environmental Protection Agency (EPA).

The complete toxicological evaluation performed by the Joint FAO/WHO Meeting on Pesticide Residues (JMPR) established an Acceptable Daily Intake (ADI) of 0.02 mg L^−1^, with conclusions of low acute toxicity and the absence of a relevant carcinogenic alert (JMPR/FAO/OMS, 2001). Safety for human health is reinforced by the EPA classification, which considers Spinosad as “not likely to be carcinogenic to humans” in its evaluations of tolerances for residues in food (EPA 2023).

Additionally, recent reviews by the European Food Safety Authority (EFSA) confirmed the toxicological parameters and indicated low acute and long-term risk for birds, ratifying the product’s suitability for multiple crop applications (EFSA 2025). Despite the favorable safety profile of the formulations containing spinosyns A and D, it is crucial to recognize the need for direct assays with the bioinputs on macroscale, particularly on non-target beneficial species. Spinosyns A and D are known to have rapid environmental degradation, especially by photolysis, contributing to their lower overall impact, but toxicity to aquatic organisms and beneficial invertebrates under field conditions still requires more in-depth verification (Cleveland et al. 2002).

### Cost-benefit analysis of bioinsecticides for pest management

Table 8 shows the costs according to the efficiency of different biological treatments for the control of *S. frugiperda*, *Dalbulus maidis*, and *Ceratitis capitata*.

**Table 8:** Costs according to the efficiency of different biological treatments for the control of *S. frugiperda*, *Dalbulus maidis*, and *Ceratitis capitata*

Target	Treatment	Dosage (L ha^−1^)	*In vitro* mortality (%)	Cost/L of product (US$)	Management cost (US$ ha^−1^)
*Spodoptera frugiperda*	*Saccharopolyspora spinosa*	7.0	82.5	1.53	10.72
	*Bacillus thuringiensis*	8.0	55.0	0.87	6.94
	*B. thuringiensis/S. spinosa*	4.0/5.0	65.0	1.14	10.29
	*Bacillus thuringiensis (Xentari)*	0.8^λ^	97.5	67.47	54.01
*Dalbulus maidis*	*Saccharopolyspora spinosa*	7.0	100.0	1.53	10.72
	*Cordyceps fumosorosea (Octane)*	0.8	80.7	39.08	31.27
*Ceratitis capitata*	*Saccharopolyspora spinosa*	10 mL L^−1μ^	64.06	1.53	10.72
	Spinetoram (25%)	5 mg L^−1μ^	92.18	255.24	51.15

^λ^Kg ha^−1^. ^μ^Dose used in the bait for the purposes of the laboratory experiment

In controlling *S. frugiperda*, *S. spinosa* stands out as a very advantageous option because it offers a mortality rate of 82.5% at a very affordable cost of US$10.72 ha^−1^, which represents a good balance between effectiveness and economy. In comparison, *B. thuringiensis* marketed as Xentari, when used individually, presents a higher mortality rate of 97.5%, but at a cost five times higher than the treatment with *S. spinosa*, which is US$54.01 ha^−1^.

Thus, *S. spinosa* may be an efficient and more economical alternative for the management of *S. frugiperda*, especially when seeking to optimize the high costs of agricultural production.

The treatment with Octane showed lower *in vitro* mortality, 72.7%, against *D. maidis* compared to *S. spinosa*, 100% for nymphs after 17 days of application. Nevertheless, this superior performance was accompanied by a lower management cost, approximately three times more compensatory, US$10.72 ha^−1^ *vs*. US$31.27 ha^−1^.

Spinetoram showed greater efficacy in the control of *C. capitata*, 92.18% compared to *S. spinosa*, 64.06%; however, its management cost was significantly higher, almost five times higher, US$51.15 ha^−1^ *vs*. US$10.72 ha^−1^. A more precise estimate of the dose required of the bioinput produced by *S. spinosa* to achieve 92.18% mortality of *C. capitata*, such as Spinetoram, based on the dose-response curve (2 mL L^−1^ and 37.5 ± 4.72; 4 mL L^−1^ and 39.06 ± 6.44; 6 mL L^−1^ and 42.18 ± 4.69; 8 mL L^−1^ and 50.00 ± 3.34; 10 mL L^−1^ and 64.06 ± 4.38) is close to 15 mL L^−1^. Even maximizing the dosage to match the mortality of the commercial product, the cost would be reduced by a factor of more than three times, US$16.07 ha^−1^, in relation to Spinetoram. Therefore, the choice between treatments must always consider the cost-benefit ratio and the level of control required.

Finally, pest control using chemical insecticides is highly effective in initial applications, especially when using formulations based on pure spinosyns A and D (Spinosad) or J and L (Spinetoram). However, under practical agricultural management conditions, a rapid decline in the efficiency of these compounds is often observed, with reductions of up to 50% after the fifth consecutive application. This decline in performance is associated with the selection of resistant populations, favored by repeated exposure to molecules with a defined and constant chemical structure. In contrast, the secondary metabolites produced by *S. spinosa*, present in bioinputs of biological origin, exhibit high complexity and structural diversity. This chemical variability makes it difficult for target pests to select resistance mechanisms since adaptation would require multiple and specific genetic mutations, which are unlikely in the short and medium term (Vontas et al. 2011). In this context, the adoption of integrated management strategies that combine chemical pesticides with biological agents has been strongly recommended in order to enhance the effectiveness of control and delay the development of resistance in pest populations while also significantly reducing agricultural production costs.

In the 2024/2025 harvest, the Brazilian bioinput market grew by 13%, maintaining an average annual expansion of 22% over the last 3 years, a rate four times higher than the global average (Gonçalves 2025). The potential treated area reached 156 million hectares, with the adoption rate per area rising from 23 to 26%, reflecting the country’s consolidation as a leader in the use of biological technologies in agriculture. Latin America is following this trend and is expected to increase its share of the global biological control market from 20% in 2021 to 29% by 2029, positioning itself as the largest regional market (CropLife Brasil 2025).

On the global stage, the bioinput market was estimated at US$14 billion in 2024, with a forecast of reaching US$20 billion by 2027, driven by the growing integration between chemical and biological pesticides. Among the segments, the highlight was bioinsecticides, whose average adoption rate increased from 26 to 30%, reflecting their effectiveness, acceptance in the field, and potential partial or total replacement for chemical insecticides. These advances reinforce the strategic role of bioinputs in increasing productivity with less environmental impact and indicate a continuous and structured expansion of the sector, with a strong protagonism of Brazil and Latin America on the global stage (CropLife Brasil 2025).

In this context, Brazil emerges as a protagonist in the use of bioinputs, with emphasis on the use of bioinsecticides mainly in soybean, corn, sugarcane, cotton, coffee, fruit and vegetables, and citrus. Technical advances, integration with pesticides, and expansion in large-scale crops indicate a scenario of sustained growth aligned with global sustainable agriculture goals.

## Conclusions

The molecular characterization of the bioinput produced by *Saccharopolyspora spinosa* revealed a rich and multifunctional composition, with emphasis on the presence of spinosyns A and D, in addition to other metabolites with antimicrobial, cytotoxic, and indirect defensive potential activity. The combination of these bioactive compounds with different mechanisms of action suggests a probable synergistic and multi-target effect, reducing the risk of resistance and increasing agronomic efficacy, as demonstrated here. Additionally, the management costs per hectare, based on its production conditions investigated here, were three to five times lower compared to commercial products, evidencing its high economic viability.

The total concentration of spinosyns A and D identified was 42.7 mg L⁻^1^, a significant value that indicates the robustness and potential for scalability of the production process adopted, since the bioinput was obtained by microbial fermentation at a strong pilot scale in 800-L bioreactors. The quantification method was effective in quantifying spinosyns A and D, with low LOD and LOQ values. In this context, this composition can provide useful information to validate the use of the bio-input and establish quality control parameters for the production protocol of new biopesticides.

In the bioassays, the bioinput demonstrated high efficacy in controlling *Spodoptera frugiperda*, with a mortality rate of 82.5% after 10 days of application, a relevant performance compared to the commercial product Xentari. Positive results were also observed against *Dalbulus maidis*, with a mortality rate of 100.0% of nymphs after 17 days, surpassing the commercial product Octane in this period, and against *Ceratitis capitata*, whose application in baits at a concentration of only 10 mL L⁻^1^ resulted in the mortality of 64% of the insects. In the latter case, it is estimated that, by increasing the dosage to 15 mL L⁻^1^, the efficacy in mortality is equivalent to the 92% obtained with 5 mg L⁻^1^ of Spinetoram at 25%. Therefore, the bioinput produced by *Saccharopolyspora spinosa* demonstrated a highly relevant chemical composition for biological pest control, with a probable synergistic effect of several molecules together with spinosyns A and D, which, at final concentrations between 0.5 and 1.5 mg L⁻^1^, showed promise in the *in vitro* control of fall armyworm, corn leafhopper, and fruit fly.

The use of *S. spinosa* bioinputs on a macroscale constitutes an accessible and viable tool for all producers, offering an effective and low-cost alternative to the chemical control of key agricultural pests, including *S. frugiperda*, *D. maidis*, and *C. capitata*. Furthermore, the study supports the development of a formulation with lower environmental risk and greater selectivity than synthetic insecticides, actively promoting integrated pest management.

These results indicate that the formulation can be safely scaled, although non-target species are amenable to future research studies, up for practical applications in agriculture, offering an effective and sustainable approach for integrated pest management, potentially partially or completely replacing synthetic insecticides in various cropping systems, thus expanding its market share in Brazil, Latin America, and the world.

## Supplementary information

Below is the link to the electronic supplementary material.

Supplementary file 1 (DOCX 1.05 MB)

## Data Availability

The data are available in the manuscript itself and in the supplementary material.

## Declarations

### Declaration of the use of AI technologies

The authors declare that they did not use AI Technologies.

## References

[CR1] Abbott WS (1925) A method of computing the effectiveness of an insecticide. J Econ Entomol 18:265–267

[CR2] Azab MM (2015) Combined activity of spinosyns A, D, J and L on two stored product insects. Egypt Acad J Biol Sci F Toxicol Pest Control 7:71–80

[CR3] Bacci L, Lupi D, Savoldelli S, Rossaro B (2016) A review of spinosyns, a derivative of biological acting substances as a class of insecticides with a broad range of action against many insect pests. J Entomol Acarol Res 48:40–52

[CR4] Baldin MM (2018) Concentration and lethal time of toxic baits based on spinosyns on *Ceratitis capitata* and *Diachasmimorpha longicaudata*. Pesqui Agropecu Trop 48:323–330

[CR5] Baronio CA, Bernardi D, Nunes MZ, Pasinato J, Garcia FRM, Botton M (2019) Bioassay method for toxicity studies of toxic bait formulations to *Ceratitis capitata* (Diptera: Tephritidae). Neotrop Entomol 48:356–363 30519927 10.1007/s13744-018-0653-0

[CR6] Basumatary D, Bailung H, Jorvekar SB, Borkar RM, Sankaranarayanan K (2023) Investigating the impact of inbuilt cold atmospheric pressure plasma on molecular assemblies of tryptophan enantiomers: in vitro fabrication of self-assembled supramolecular structures. RSC Adv 13:26640–26649 37681043 10.1039/d3ra04086k PMC10480704

[CR7] Block MA, Dorne AJ, Joyard J, Douce R (2007) Probing Arabidopsis chloroplast diacylglycerol pools by selectively targeting diacylglycerol kinase to the envelope membrane. Plant Cell 19:326–340

[CR8] Borges R, Machota R Jr, Boff MIC, Botton M (2015) Effect of toxic baits on Anastrepha fraterculus (Wiedemann) (Diptera: Tephritidae). BioAssay 10:134

[CR9] Braga AFV, Rosário MSD, Gomes JBN, Monteiro CDA, Farias FA, Rodrigues Filho E, Cantanhede Filho AJ (2024) Antimicrobial potential of soil/sediment mangrove associated fungi: a review. J Braz Chem Soc 35:e-20240032

[CR10] Bravo A, Soberón M, Gómez I (2020) Cry1Ac protoxin and its activated toxin from Bacillus thuringiensis act differentially during the pathogenic process. J Agric Food Chem 68:5816–5824 32379448 10.1021/acs.jafc.0c01172

[CR11] Chen L, Liu W, Hu X, Huang K, Wu JL, Zhang QQ (2011) Citrinin derivatives from the marine-derived fungus *Penicillium citrinum*. Chem Pharm Bull 59:515–520 10.1248/cpb.59.515 21467687

[CR12] Chen J, Xia H, Dang F, Xu Q, Li W, Qin Z (2015) Characterization of the chromosomal integration of *Saccharopolyspora* plasmid pCM32 and its application to improve production of spinosyn in *Saccharopolyspora spinosa*. Appl Microbiol Biotechnol 99:10141–10149 26260388 10.1007/s00253-015-6871-z

[CR13] Chet I, Inbar J (1994) Biological control of fungal pathogens. Appl Biochem Biotechnol 48:37–43 7979350 10.1007/BF02825358

[CR14] Choub V, Maung CEH, Won S-J, Park S-H, Ahn Y-S (2021) Antifungal activity of cyclic tetrapeptide from Bacillus velezensis CE 100 against plant pathogen Colletotrichum gloeosporioides. J Agric Food Chem 69:1255–1262 10.3390/pathogens10020209 PMC7919652 33672094

[CR15] Cleveland CB, Mayes MA, Cryer SA (2002) An ecological risk assessment for Spinosad use on cotton. Pest Manag Sci 58:70–84 11838288 10.1002/ps.424

[CR16] CropLife Brasil (2025) Adoption of bioinputs grew 13% in the 2024/2025 crop year. CropLife Brasil. https://croplifebrasil.org/adocao-de-bioinsumos-cresceu-13-na-safra-2024-2025/. Accessed 7 Jun 2025

[CR17] Díaz SA, Leal GN, Silva NF, Venâncio MGS, Figueiredo KG, Oliveira JAC, Lopes NJ (2024) *Spodoptera frugiperda*: a significant threat to Brazilian agriculture. Revista Cultivar. https://revistacultivar.com/articles/Spodoptera-frugiperda-is-a-significant-threat-to-Brazilian-agriculture. Accessed 27 Jun 2025

[CR18] EFSA (2025) Conclusion on the peer review of the pesticide risk assessment of the active substance Spinosad. EFSA J 23(4):e9193 39834753 10.2903/j.efsa.2025.9193 PMC11744298

[CR19] EPA (2023) Spinosad. Pesticide tolerances. Fed Regis 88(165):58632–58640

[CR20] Faria M, Santos MA, Santos W, Rodrigues B, Andrade Z (2023) On-farm production of microbial entomopathogens for use in agriculture: Brazil as a case study. Neotrop Entomol 52:122–133 37014592 10.1007/s13744-023-01033-5

[CR21] Ferreira DF (2000) Statistical analyses using SISVAR (System for Analysis of Variance) for Windows 4.0. In: Proceedings of the 45th nnual Meeting of the Brazilian Region of the International Biometric Society. UFSCar, São Carlos, pp 255–258

[CR22] Ferreira EB, Cavalcanti PP, Nogueira DA (2014) ExpDes: an R package for ANOVA and experimental designs. Appl Math 5:2952–2958

[CR23] Flores S, Gomez LE, Montoya P (2011) Residual control and lethal concentrations of GF-120 (Spinosad) for Anastrepha spp. (Diptera: Tephritidae). J Econ Entomol 104:1885–1891 22299349 10.1603/ec10365

[CR24] Gonçalves F (2025) The evolution of bioinputs: quality makes the difference. Globo Rural. https://globorural.globo.com/opiniao/noticia/2025/05/a-evolucao-dos-bioinsumos-qualidade-faz-a-diferenca.ghtml. Accessed 5 Jun 2025

[CR25] Gonçalves EP, Cruz I, Figueiredo MLC, Ciociola AI (1997) Effect of ‘Spinosad’ on Spodoptera frugiperda Smith larvae and its natural enemies, the predator Doru luteipes Scudder and the parasitoid Campoletis flavicincta Ashmead. In: 16th Brazilian Congress of Entomology, Salvador. Abstracts. Salvador: SEB/EMBRAPA-CNPMF, p 177

[CR26] Gopalakrishnan S, Rajendran V, Arumugam S, Sharma HC, Vadlamudi S, Bhimineni RK, Simic N (2016) Insecticidal activity of a novel fatty acid amide derivative from Streptomyces species against Helicoverpa armigera. Nat Prod Res 30:2760–2769 26956775 10.1080/14786419.2016.1154055

[CR27] Gotthardt A, Bury D, Kling H-W, Weiss T, Belov V, Qi Y, Koch HM (2019) Determination of human urinary metabolites of the plasticizer di(2-ethylhexyl) adipate (DEHA) by online-SPE-HPLC-MS/MS. J Chromatogr B 1124:239–246 10.1016/j.jchromb.2019.06.019 31233945

[CR28] Hafez AM, Abbas N (2021) Insecticide resistance to insect growth regulators, avermectins, spinosyns, and diamides in *Culex quinquefasciatus* in *Saudi Arabia*. Parasit Vectors 14:1–9 34715900 10.1186/s13071-021-05068-8 PMC8555291

[CR29] Hezakiel HE, Thampi M, Rebello S, Sheikhmoideen JM (2024) Biopesticides: a green approach towards agricultural pests. Appl Biochem Biotechnol 196:5533–5562 37994977 10.1007/s12010-023-04765-7

[CR30] Huang K, Xia L, Zhang Y, Ding X, Zahn JA (2009) Recent advances in the biochemistry of spinosyns. Appl Microbiol Biotechnol 82:13–23 19082588 10.1007/s00253-008-1784-8

[CR31] Jha AK, Jang JP, Shin YK, Lee BR, Cho JH, Choi YJ (2014) Metabolic engineering of rational screened Saccharopolyspora spinosa for the enhancement of spinosyns A and D production. Mol Cells 37:727–733 25256218 10.14348/molcells.2014.0168 PMC4213763

[CR32] Jin ZH, Wang F, Li H, Chen Z (2009) Enhanced production of Spinosad in *Saccharopolyspora spinosa* by genome shuffling. Appl Biochem Biotechnol 159:655–663 19132553 10.1007/s12010-008-8500-0

[CR33] JMPR/FAO/OMS (2001). Spinosad. In: Pesticide Residues in Food - 2001, Toxicological Evaluations. Joint Meeting of the FAO Panel of Experts on Pesticide Residues in Food and the Environment and the WHO Core Assessment Group (JMPR). Available at https://www.inchem.org/documents/jmpr/jmpmono/2001pr12.htm

[CR34] Kumar D, Singh A, Sharma P, Gupta R (2023) Fungal biofertilizers and biopesticides and their roles in sustainable agriculture. In: Gupta VK (ed) Applied Mycology for Agriculture and Foods. Apple Academic Press, Palm Bay, FL, pp 165–216

[CR35] Madduri K, Waldron C, Merlo DJ (2001a) Rhamnose biosynthesis pathway supplies precursors for primary and secondary metabolism in Saccharopolyspora spinosa. J Bacteriol 183:5632–5638 11544225 10.1128/JB.183.19.5632-5638.2001 PMC95454

[CR36] Madduri K, Zirkle RE, Jaskulka BT (2001b) Genes for the biosynthesis of spinosyns: applications for yield improvement in Saccharopolyspora spinosa. J Ind Microbiol Biotechnol 27:399–402 11774006 10.1038/sj.jim.7000180

[CR37] Marcinkevicius K, Salvatore SA, Bardon ADV, Cartagena E, Arena ME, Vera NR (2017) Insecticidal activities of diketopiperazines of *Nomuraea rileyi* entomopathogenic fungus. Int J Environ Agric Biotechnol 2:1586–1596

[CR38] Markham JE, Lozano-Rosas MG, Dietrich CR, Ramos-Vega M, Cahoon EB, Gavilanes-Ruíz M (2011) MPK6, sphinganine and the LCB2a gene from serine palmitoyltransferase are required in the signaling pathway that mediates cell death induced by long chain bases in Arabidopsis. New Phytol 190:487–499 10.1111/j.1469-8137.2011.03727.x 21534970

[CR39] Mertz FP, Yao RC (1990) *Saccharopolyspora spinosa* sp. nov. isolated from soil. Int J Syst Bacteriol 40:223–228 10.1099/00207713-40-1-28 2223595

[CR40] Nunes MZ, Bernardi D, Baronio CA, Pasinato JA (2019) A laboratory bioassay method to assess the use of toxic bait on *Anastrepha fraterculus* (Weidemann 1830). Neotrop Entomol. 10.1007/s13744-019-00728-y 31741220 10.1007/s13744-019-00728-y

[CR41] Okazaki Y, Saito K (2018) Roles of lipid metabolism in plant defense. J Agric Food Chem 66:3947–3952

[CR42] Ongena M, Jacques P (2008) *Bacillus* lipopeptides: versatile weapons for plant disease biocontrol. Trends Microbiol 16:115–125 18289856 10.1016/j.tim.2007.12.009

[CR43] Püntener W, Zahner O (1981) Manual for field trials in plant protection, 2nd edn, rev. and enl. Ciba-Geigy, Basle

[CR44] Rahman MM, El-Aty AMA, Ara J, Park J-H, Kim M-R, Eun J-B, Shin H-C, Shim J-H (2021) Quantification of spinosyn A and spinosyn D in animal-derived products using multiwalled carbon nanotubes coupled with LC–MS/MS for analysis. Biomed Chromatogr 35:e5007 33067857 10.1002/bmc.5007

[CR45] Rocha TM, Dias AHS, Lins H, Goulart C, Ribeiro G, Magalhães M, Oliveira A (2024) Agricultural bioinputs obtained by solid-state fermentation: from production in biorefineries to sustainable agriculture. Sustainability 16:1076

[CR46] Rodrigues PS, dos Santos TAT, DaMatta RA, Seabra SH (2018) Evaluation of the exposure of phosphatidylserine (PS) by Plasmodium chabaudi. In: Proceedings of the 3rd Fluminense Congress of Post-Graduation. Instituto Federal de Educação, Ciência e Tecnologia (IFF), UENF, Campos dos Goytacazes. https://editoraessentia.iff.edu.br/index.php/CONPG/article/view/13285/10643. Accessed 27 Jun 2025

[CR47] Rolim GG, Guedes RNC, Siqueira HAA (2019) Susceptibility of cotton boll weevil (Coleoptera: Curculionidae) to spinosyns. J Econ Entomol 112:1688–1694 30927546 10.1093/jee/toz066

[CR48] Sarkar T, Nandi S, Bhunia D, Ghosh S (2021) Menthyloxy carbonyl valine as a plant defense inducer: a new molecule for crop protection. ACS Omega 6:4289–429733644550

[CR49] Saucedo-García M, Guevara-García A, González-Solís A, Cruz-García F, Vázquez-Santana S, Markham JE, Lozano-Rosas MG, Dietrich CR, Ramos-Vega M, Cahoon EB, Gavilanes-Ruíz M (2011) MPK6, sphinganine and the LCB2a gene from serine palmitoyltransferase are required in the signaling pathway that mediates cell death induced by long chain bases in Arabidopsis. New Phytol 190:487–499 10.1111/j.1469-8137.2011.03727.x 21534970

[CR50] Schütze IX, Baronio CA, Baldin MM, Loek AE, Botton M (2018) Toxicity and residual effects of toxic baits with spinosyns on the South American fruit fly. Pesqui Agropecu Bras 53(02):144–151

[CR51] Sun R, Zhang J, Yin L, Pu Y (2014) Investigation into variation of endogenous metabolites in bone marrow cells and plasma in C_3_H/He mice exposed to benzene. Int J Mol Sci 15:4994–5010 24658442 10.3390/ijms15034994 PMC3975436

[CR52] Tanaka N, Steiner LF, Ohinata K, Okamoto R (1969) Low-cost larval rearing medium for mass production of oriental and Mediterranean fruit flies. J Econ Entomol 62:967–968

[CR53] Thompson GD, Cleveland CB, McCormick KL (2000) Spinosad-a case study: an example from a natural products discovery programme. Pest Manag Sci 56:660–664

[CR54] Timm S, Florian A, Arrivault S, Stitt M, Fernie AR, Bauwe H (2012) Glycine decarboxylase controls photosynthesis and plant growth. Plant Cell 24:588–606 10.1016/j.febslet.2012.08.027 22982108

[CR55] Tomquelski GV, Martins GLM (2007) Eficiência de inseticidas sobre *Spodoptera frugiperda* (J.E. Smith, 1797) (Lepidoptera: Noctuidae) em milho na região dos Chapadões. Rev Bras Milho Sorgo 6:26–39

[CR56] Vassilakos TN (2023) Spinetoram: a potential grain protectant. Crop Prot 173:106354

[CR57] Vassilakos TN, Athanassiou CG, Kavallieratos NG, Tomanović Ž (2012) Insecticidal effect of Spinetoram against six major stored grain insect species. J Stored Prod Res 51:69–73

[CR58] Vontas J, Hernández-Crespo P, Van Leeuwen T, Ranson H (2011) Insecticide resistance in Tephritid flies. Pestic Biochem Physiol 100:199–205

[CR59] Wada H, Murata N (2009) Lipids in photosynthesis: essential roles in photosystem II and photosystem I. Biochim Biophys Acta 1787:288–296

[CR60] Wang SC, Chen W, Liu Y, Xu M, Wang J, Lin L, Zhang J (2016) Matrix-assisted laser desorption/ionization mass spectrometry imaging of cell cultures for the lipidomic analysis of potential lipid markers in human breast cancer invasion. Rapid Commun Mass Spectrom 30:533–542 26777684 10.1002/rcm.7466

[CR61] Wang W, Lu Z, Zhang J, Liu Q, Wang L, Wang H (2020) Lipid signaling in plant defense: roles of phosphatidylethanolamine and derivatives. J Agric Food Chem 68:3785–3793

[CR62] Xu Z, Zhang B, Liu X, Wang Q, Lin Y, Wu R, Ma R, Wang J, Lu X (2024) Breeding of butenyl-spinosyns high yielding strain by 60Co-NTG compound mutation and its insecticidal activity. Chin J Biol Control 2:299

[CR63] Yamamoto PT, Müller C, Bedendo IP (2020) Efficacy of insecticides in the control of Dalbulus maidis (DeLong & Wolcott) (Hemiptera: Cicadellidae) and the transmission of corn spiroplasma. PhD Thesis, University of São Paulo (USP), Piracicaba

[CR64] Yang X, Niu J, Guo X (2014) Genome-scale metabolic network reconstruction of Saccharopolyspora spinosa for Spinosad production improvement. BMC Syst Biol 8:1–13 24628959 10.1186/1475-2859-13-41 PMC4003821

[CR65] Yang G, Cao S, Yan J, Wang Q, Ma R, Lu X (2016) A new medium for improving Spinosad production by *Saccharopolyspora spinosa*. Jundishapur J Microbiol 9:e16765 27635207 10.5812/jjm.16765 PMC5013548

[CR66] Yang S-Q, Li X-M, Li X, Li H-L, Meng L-H, Wang B-G (2018) New citrinin analogues produced by coculture of the marine algal-derived endophytic fungal strains Aspergillus sydowii EN-534 and Penicillium citrinum EN-535. Phytochemistry Lett 25:191–195

[CR67] Yao G, Sebisubi FM, Voo LYC, Ho CC, Tan GT, Chang LC (2011) Citrinin derivatives from the soil filamentous fungus Penicillium sp. H9318. J Braz Chem Soc 22:1125–1129

[CR68] Ye H, Zhang Y, Chen Y, Li J, Zhang H, Luo Y, Pan J (2020) ATR-FTIR spectroscopic identification of red blood cell changes induced by sepsis. Analyst 145:5311–5319

[CR69] Zhang Y, Liu X, Yin T, Li Q, Zou Q, Huang K, Zhang X (2021) Comparative transcriptomic analysis of two *Saccharopolyspora spinosa* strains reveals the relationships between primary metabolism and Spinosad production. Sci Rep 11:14779 34285307 10.1038/s41598-021-94251-z PMC8292330

[CR70] Zuo Y, Wu J, Li M, Zhang X, Wang H (2020) Functional validation of nicotinic acetylcholine receptor (nAChR) α6 as a target of spinosyns in *Spodoptera exigua* utilizing the CRISPR/Cas9 system. Pest Manag Sci 76:2415–2422 32056365 10.1002/ps.5782

[CR71] Zuo Y-Y, Li M, Wang L, Zhang X, Liu H (2022) Knockin of the G275E mutation of the nicotinic acetylcholine receptor (nAChR) α6 confers high levels of resistance to spinosyns in *Spodoptera exigua*. Insect Sci 29:478–486 33998150 10.1111/1744-7917.12922

