# Supplementary material for: Chemical composition and bioinsecticidal activity of bioinputs produced by Saccharopolyspora spinosa
Source: Appl Microbiol Biotechnol. 2025 Dec 11;109:267. doi: 10.1007/s00253-025-13655-3 (PMC12701044; doi:10.1007/s00253-025-13655-3)
Supplement: Supplementary file 1 [file 253_2025_13655_MOESM1_ESM.docx]

**Supporting Information**

Spectra obtained by high-resolution HPLC-MS of samples aged 15, 90 and 180 days.

(+)-ESI-MS Valine (*m/z* 118.0858 [M – H]^–^).

(-)-ESI-MS Glyceric acid (*m/z* 151.0238 [M – H]^–^).

(+)-ESI-MS of Leucine (*m/z* 132.1015 [M + H]^+^).

(+)-ESI-MS of Phenylalanine (*m/z* 166.088 [M + H]^+^).

(+)-ESI-MS of Tryptophan (*m/z* 205.0977 [M + H]^+^).

(-)-ESI-MS Unknow I (*m/z* 181.0444 [M – H]^–^).

(+)-ESI-MS of Cyclo(Met-Val) (*m/z* 231.1150 [M + H]^+^).

(+)-ESI-MS of N-stearoyl tryptophan (*m/z* 453.3463 [M + H]^+^).

(+)-ESI-MS of Sphinganine (*m/z* 340.2618 [M + H]^+^).

(+)-ESI-MS Unknown II (*m/z* 396.8039 [M + H]^+^).

(-)-ESI-MS 3-hydroxyhexanoic acid (*m/z* 131.0715 [M – H]^–^).

(-)-ESI-MS 3-hydroxy-3-phenylpropionic acid (*m/z* 165.0544 [M – H]^–^).

(+)-ESI-MS of Spinosyn A (*m/z* 732.4694 [M + H]^+^).

(-)-ESI-MS 2-[(*E*)-5-methyl-3-oxododec-4-enimidoyl]oxyhexanoic acid

(*m/z* 384.2411 [M + HCOOH]^–^).

(+)-ESI-MS of Spinosyn D (*m/z* 746.4833 [M + H]^+^).

(-)-ESI-MS Unknow III (*m/z* 204.0602 [M+HCOOH]^–^).

(+)-ESI-MS Penicitrinol D (*m/z* 279.1599 [M + H]^+^).

(-)-ESI-MS PG(O-18:0/0:0) (*m/z* 497.3246 [M+HCOOH]^–^).

(+)-ESI-MS Cyclo-(L-Ile-L-Leu-L-Leu-L-Leu-L-Leu) (*m/z* 566.4270 [M+H^+^).

(-)-ESI-MS PE(O-20:0/4:0) (*m/z* 610.4077 [M+HCOOH]^–^).

(+)-ESI-MS DG(i-17:0/0:0/20:4(7E,9E,11Z,13E)-3OH(5S,6R,15S)) (*m/z* 701.4921 [M + Na]^+^).

(+)-ESI-MS of Diethylhexyl adipate (*m/z* 371.3155 [M + H]^+^).

(-)-ESI-MS PA(18:0/20:4) (*m/z* 723.4899 [M – H]^–^).

(+)-ESI-MS PC(P-18:1(9Z)/20:4(5Z,8Z,11Z,14Z)) (*m/z* 814.5768 [M + Na]^+^).

(-)-ESI-MS PS (16:0/20:0) (*m/z* 836.5683 [M – H]^–^).

(-)-ESI-MS Unknow IV (*m/z* 949.6493 [M – H]^–^).
